# HEART Score Agreement Between Attending and Resident Emergency Medicine Physicians for Patients With Potential Acute Coronary Syndrome

**DOI:** 10.31486/toj.24.0108

**Published:** 2025

**Authors:** Joel C. Mosley, Greggory R. Davis, Michael H. Truax

**Affiliations:** ^1^ Emergency Medicine, University of Arkansas for Medical Sciences, Little Rock, AR; ^2^ Our Lady of the Lake Regional Medical Center, Baton Rouge, LA; ^3^ Emergency Medicine, Louisiana State University Health Sciences School of Medicine, Baton Rouge, LA

**Keywords:** *Emergency medicine*, *heart arrest*, *reproducibility of results*, *risk assessment*

## Abstract

**Background:**

Chest pain in the emergency department requires swift diagnosis to distinguish between acute coronary syndrome and noncardiac causes. The use of the HEART score, which risk-stratifies patients based on history, electrocardiogram, age, risk factors, and troponin, reduces unnecessary admissions and costs. However, evaluations by resident physicians supervised by attending physicians can delay treatment and increase costs.

**Methods:**

We assessed interrater reliability between attending physician and resident physician HEART scores in 2 study phases. In phase 1, participants were not provided with a standardized form, but in phase 2, participants used a standardized form to calculate HEART scores. Differences in scores were compared by years of experience and by study phase.

**Results:**

A total of 75 HEART score comparisons were analyzed. Fifty comparisons between attending physicians and resident physicians were completed in phase 1, and 25 comparisons were completed in phase 2. Discrepancies between attending and resident physician scores ≤3 vs >3 decreased from 24% in phase 1 to 8% in phase 2. Attending physician years of experience did not affect discrepancies in HEART scores ≤3 vs >3 between attending and resident physicians (odds ratio [OR] 1.18 [95% CI 0.78 to 1.81]). Similarly, resident physician years of experience did not affect differences in HEART scores ≤3 vs >3 between attending and resident physicians (OR 0.77 [95% CI 0.38 to 1.53]).

**Conclusion:**

The study found good agreement between attending physician and resident physician HEART scores, with experience level not significantly affecting discrepancies. The standardized scoring form improved consistency, although not significantly.

## INTRODUCTION

Chest pain is a common cause of visits to the emergency department (ED) and can originate from a variety of cardiac and noncardiac diseases. The ability to make a quick and definitive diagnosis that distinguishes chest pain caused by acute coronary syndrome from a noncardiac etiology is paramount in the ED setting.^1^ Since the HEART score system was developed in 2008,^1^ it has been widely adopted in EDs to quickly and accurately diagnose acute coronary syndrome. The HEART score is a risk stratification of the 5 factors that form the acronym: history, electrocardiogram (ECG), age, risk factors, and troponin. Each factor is scored from 0 to 2, and the highest possible HEART score is 10. A HEART score ≤3 indicates that a patient is at low risk for a major adverse cardiac event and is thus deemed safe for discharge from the ED without provocative testing after discharge.^1-3^

The effectiveness of using the HEART score in the ED has been demonstrated in various studies. A 2010 study by Backus et al validated the HEART score in a multicenter cohort, showing its ability to accurately stratify patients with chest pain into different risk categories for major adverse cardiac events, thus aiding in clinical decision-making and reducing unnecessary admissions and diagnostic testing.^2^ Similarly, use of the HEART Pathway, which includes the use of the HEART score, has been shown to reduce cardiac testing, hospital admissions, and hospital length of stay and to increase early discharge without compromising patient safety.^4,5^ In 2021, Bylund et al reported that the implementation of a standardized protocol for chest pain evaluation, which included the HEART score, significantly reduced the length of stay in the ED and overall hospital costs.^6^ Additionally, a 2021 review by Ashburn et al highlighted that multiple studies have found that the HEART Pathway can be used to effectively triage patients, leading to a reduction in unnecessary hospital admissions and associated costs.^7^

In academic medical center EDs, a patient is first evaluated by a resident physician, and the findings of the history and physical, including the HEART score, are discussed with a supervising attending physician. This process often leads to a longer patient evaluation time, which is not only unsafe for high-risk patients but also leads to an increase in length of stay and total hospital costs for patients who are deemed low risk. Studies have shown that prolonged evaluation times and increased length of stay are associated with higher health care costs and resource utilization.^8-10^

The primary aim of this study was to determine if the tabulation of HEART scores by emergency medicine resident physicians agreed with independently derived attending physician HEART scores. If good interrater reliability exists, attending physicians may be able to rely on resident physicians’ HEART scores. Attending physicians would thus be able to dedicate time to other areas of need in the busy ED. The secondary aim of this study was to determine if a standardized HEART score form could improve interrater reliability between attending and resident physicians.

## METHODS

### Study Design

This study involved the prospective collection of data on emergency medicine attending and resident physicians at a regional medical center in the southeastern United States with the goal of determining interrater reliability between attending and resident HEART score calculations in the ED.

### Study Procedures

Prior to the start of phase 1, attending and resident physicians were presented with an explanation of the study and rationale for the study. All physicians were given a study information sheet during a resident education conference. Along with the purpose of the study, the information sheet stated that no identifying information would be collected, and consent was implied through the completion of the study forms. The study was approved by the local institutional review board.

During phase 1, study personnel identified potential scenarios for which a HEART score would be calculated by watching the ED tracking board. When a scenario was identified, study personnel approached the attending and resident working on that case and provided them with envelopes containing a sheet of paper with the question, “What is the HEART score for this patient?” Attending and resident physicians anonymously determined and recorded the HEART score, and their responses were linked by consecutive, anonymous subject numbers (eg, HEART1_attending and HEART1_resident were linked). Attending physicians were asked to record their experience level according to the following ranges: 0 to 2, 3 to 5, 6 to 10, or >10 years. Resident physicians were asked to record their postgraduate year. After providing the HEART score and their experience level, attending and resident physicians were asked to put the form in the envelope, seal it, and return it to the study personnel who then placed the envelopes in a locked box in the ED. After they submitted their answers, the attending and resident physicians were allowed to confer about the HEART score; however, the forms were completed independently prior to submission.

**Figure 1.**
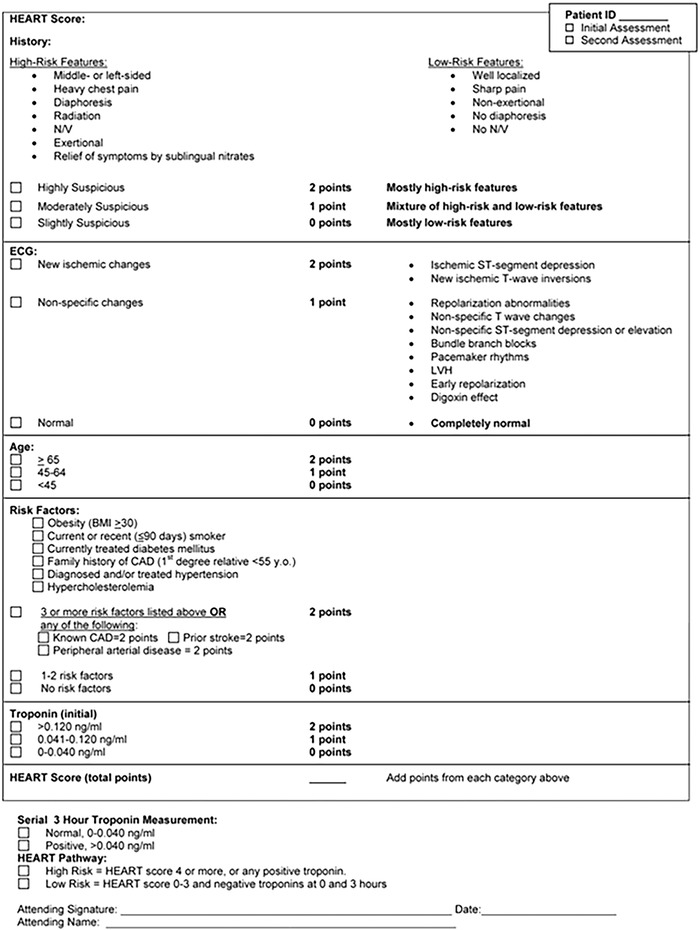
**A HEART score standardized form derived from Six et al^1^ was used in phase 2 of the study.** BMI, body mass index; CAD, coronary artery disease; HEART, history, electrocardiogram, age, risk factors, troponin; LVH, left ventricular hypertrophy; N/V, nausea/vomiting; y.o., years old.

During phase 2 of the study, the phase 1 process was repeated. However, a standardized HEART score form (Figure 1) derived from Six et al^1^ was included in the envelope to help attending and resident physicians determine HEART scores.

Data were collected between March 2018 and December 2019. Phase 1 spanned 14 months, from March 2018 through May 2019, and phase 2 spanned 6 months, from June 2019 through December 2019. The original plan for the study was to collect data for an equal amount of time in each phase. However, data collection was stopped prematurely in phase 2 because of coronavirus disease 2019.

### Statistical Analysis

Statistical analyses were performed using R version 4.2.2 (The R Foundation). Descriptive data are presented as counts and proportions. The interrater reliability between the attending and resident physician dichotomous HEART scores (≤3 or >3) was calculated with the Cohen kappa statistic. A kappa value of 0 to 0.20 was considered slight, 0.21 to 0.40 was considered fair, 0.41 to 0.60 was considered moderate, and 0.61 to 0.80 was considered substantial.^11^ Logistic regression was used to determine the effects of study phase (with or without the use of the standardized scoring form) and of years of experience on discrepancies of ≤3 or >3 in HEART scores between attending and resident physicians. Heatmap and subscore difference figures were created with the ggplot2 package^12^ in RStudio (Posit Software, PBC).

## RESULTS

A total of 77 patient HEART score comparisons, or 154 score forms, were initially evaluated. However, an attending physician HEART score was missing for 1 patient, and a component of the HEART score was missing from both the attending and resident physician for another patient that prevented direct score comparisons for 2 patients. Therefore, 75 HEART score comparisons, or 150 HEART scores, were included in the final data analysis.

Descriptive data regarding years of experience for attending and resident physicians are provided in Table 1. The frequencies for each HEART score are shown for attending and resident physicians in Table 2.

**Table 1.** Attending Physician and Resident Physician Experience Levels

Attending Physician Experience Level, years	Attending Physicians, n=75	Resident Physician Experience Level, years	Resident Physicians, n=75
0-2	22 (29.3)	PGY1	7 (9.3)
3-5	19 (25.3)	PGY2	30 (40.0)
6-10	20 (26.7)	PGY3	37 (49.3)
>10	12 (16.0)	PGY4+	1 (1.3)
Missing	2 (2.7)		

Note: Data are presented as n (%). Experience levels that were not recorded are reported as missing.

PGY, postgraduate year.

**Table 2.** Attending Physician vs Resident Physician HEART Scores

HEART Score	Attending Physician Frequency	Resident Physician Frequency
0	2 (2.7)	0
1	6 (8.0)	7 (9.3)
2	8 (10.7)	8 (10.7)
3	11 (14.7)	8 (10.7)
4	16 (21.3)	16 (21.3)
5	14 (18.7)	21 (28.0)
6	12 (16.0)	8 (10.7)
7	3 (4.0)	6 (8.0)
8	2 (2.7)	0
9	1 (1.3)	1 (1.3)
Total	75 (100)	75 (100)

Notes: From the original 77 HEART score forms collected, an attending physician HEART score was missing for 1 patient, and a component of the HEART score was missing from both the attending and resident physician for another patient that prevented direct score comparisons for 2 patients. This table reflects only complete comparisons (n=75 per group). Data are presented as n (%).

HEART, history, electrocardiogram, age, risk factors, troponin.

In phase 1 of the study, 100 HEART scores (66.7% of all scores recorded), or 50 comparisons between attending and resident physicians, were completed. In phase 2 of the study, 50 HEART scores (33.3%), or 25 comparisons, were completed.

**Figure 2.**
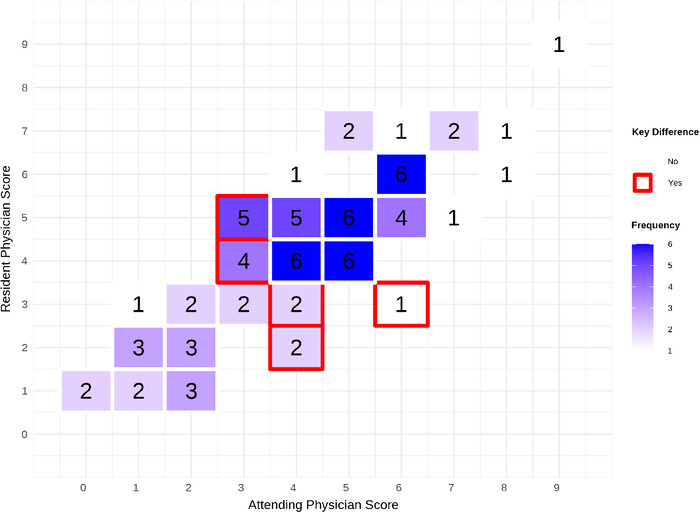
**Heatmap of attending physician vs resident physician HEART scores. The y-axis represents resident HEART scores, and the x-axis represents attending HEART scores. For each patient, the HEART scores by the attending and the resident were tallied on the heatmap (eg, a HEART score of 2 by the attending and 2 by the resident were tallied as 1 comparison in the box corresponding to 2 on the y-axis and 2 on the x-axis). The number inside each box represents the frequency of those comparisons. More frequent occurrences are represented by darker boxes, and less frequent occurrences are represented by lighter boxes. Key differences, depicted by red-bordered boxes, indicate instances in which the HEART score was ≤3 for the attending and >3 for the resident, or vice versa. Any box without a border did not fall into this category.** HEART, history, electrocardiogram, age, risk factors, troponin.

Differences in HEART scores for both phases of the study (attending score – resident score) are provided in heatmap format in Figure 2 to visualize score discrepancies. The Cohen kappa statistic was used to quantify the level of agreement between the attending and resident physicians and was found to be 0.26 (95% CI 0.12 to 0.39), representing fair agreement. Of the 75 HEART score comparisons, 14 (18.7%) had a discrepancy in which the attending scored >3 and the resident scored ≤3 or the attending scored ≤3 and the resident scored >3.

**Figure 3.**
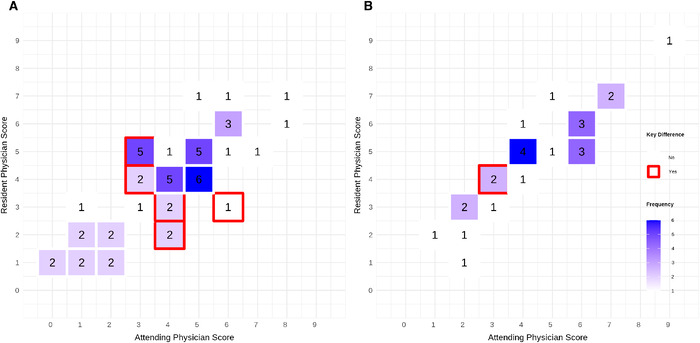
**Heatmap of attending physician vs resident physician HEART scores for (A) phase 1 and (B) phase 2 of the study. The y-axis represents resident HEART scores, and the x-axis represents attending HEART scores. For each patient, the HEART scores by the attending and the resident were tallied on the heatmap (eg, a HEART score of 2 by the attending and 2 by the resident were tallied as 1 comparison in the box corresponding to 2 on the y-axis and 2 on the x-axis). The number inside each box represents the frequency of those comparisons. More frequent occurrences are represented by darker boxes, and less frequent occurrences are represented by lighter boxes. Key differences, depicted by red-bordered boxes, indicate instances in which the HEART score was ≤3 for the attending and >3 for the resident, or vice versa. Any box without a border did not fall into this category.** HEART, history, electrocardiogram, age, risk factors, troponin.

Differences in HEART scores by phase are shown in Figure 3. The Cohen kappa statistic for phase 1 was 0.23 (95% CI 0.07 to 0.38) and 0.31 (95% CI 0.08 to 0.50) for phase 2, both representing fair agreement. Of the 50 HEART score comparisons in phase 1, 12 (24.0%) had a discrepancy in which the attending scored >3 and the resident scored ≤3 or the attending scored ≤3 and the resident scored >3. Of the 25 HEART score comparisons in phase 2, 2 (8.0%) had a discrepancy in which the attending scored >3 and the resident scored ≤3 or the attending scored ≤3 and the resident scored >3.

**Figure 4.**
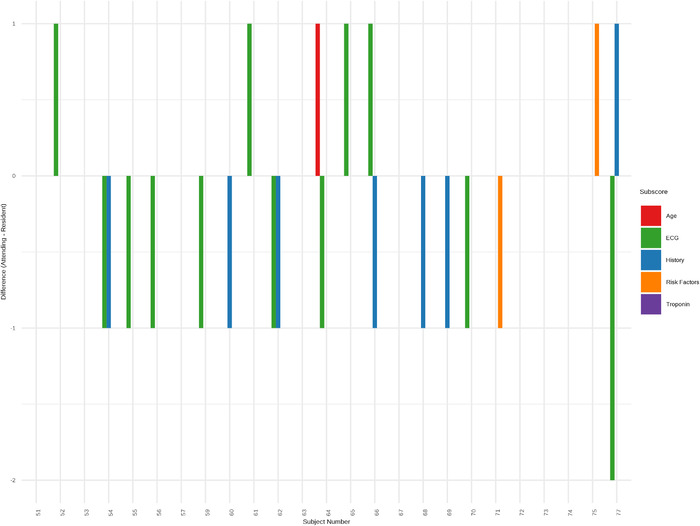
**HEART subscore differences for phase 2 of the study. For each component of the overall HEART score, the attending physician score was subtracted from the resident physician score. The x-axis shows the subject numbers of the patients who were scored. The y-axis represents the absolute difference in the subscores. Each subscore is coded by a unique color.** ECG, electrocardiogram. HEART, history, electrocardiogram, age, risk factors, troponin.

Individual subscores, or components of the HEART score, could not be compared for phase 1 of the study because only a composite HEART score was recorded. For phase 2, we were able to directly compare subscores for each patient, and the comparison is provided in Figure 4. ECG scores were the most common discrepancy, followed by history. In both cases, residents tended to score higher in these categories. History was scored higher by residents in 6 cases (24.0%) and ECG was scored higher by residents in 7 cases (28.0%). In 1 instance, the ECG score varied by more than 1 point.

Given the importance of HEART scores ≤3, this criterion was used to create a dichotomous outcome (HEART scores ≤3 or >3). Table 3 provides the odds ratios (ORs) and 95% CIs for a logistic regression with the dichotomous HEART score difference as the outcome variable, and study phase, attending years of experience, and resident years of experience as the predictor variables. The 95% CI for the OR suggests that the difference between the 24.0% discrepancy in phase 1 and the 8.0% discrepancy in phase 2 for dichotomous HEART score outcomes was not significant (OR 0.25, 95% CI <0.01 to 1,031.89). This wide CI is likely attributable to the small sample size and the large variability in the data from phase 2 and therefore should be interpreted with caution. Similarly, the *z* score for the comparison of the phase 1 and phase 2 kappa values was relatively small (–0.54, *P*=0.59), suggesting no statistical improvement in interrater reliability from phase 1 to phase 2.

**Table 3.** Logistic Regression for HEART Scores ≤3 or >3

Predictor	Odds Ratio	95% Confidence Interval
Study phase	0.25	<0.01-1,031.89
Attending physician years of experience	1.18	0.78-1.81
Resident physician years of experience	0.77	0.38-1.53

HEART, history, electrocardiogram, age, risk factors, troponin.

Attending physician years of experience was not significant in the logistic regression analysis of HEART scores ≤3 or >3, meaning that years of experience did not affect discrepancies in HEART scores between attending and resident physicians (OR 1.18, 95% CI 0.78 to 1.81). Similarly, resident years of experience did not significantly affect differences in HEART scores ≤3 or >3 (OR 0.77, 95% CI 0.38 to 1.53).

**Figure 5.**
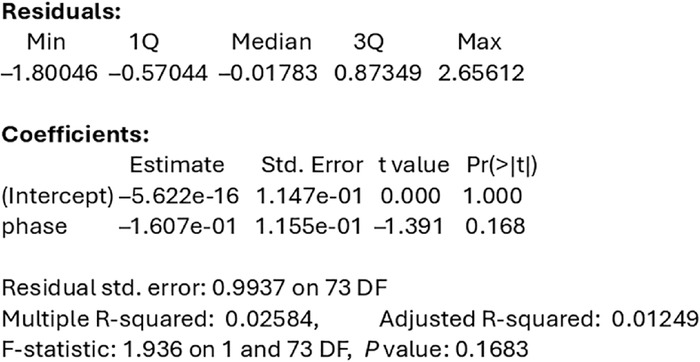
**Standardized linear regression assessing the relationship between HEART score differences and study phase (phase 1 vs phase 2).** HEART, history, electrocardiogram, age, risk factors, troponin.

If the differences in HEART scores were treated as continuous variables and a linear regression was utilized, the phase of the study (phase 1 with no standardized scoring form vs phase 2 with a standardized scoring form) did not significantly affect differences in HEART scores between attending and resident physicians despite the improved kappa values from phase 1 to phase 2 (0.23 to 0.31, respectively). The standardized beta coefficient for this model was –0.16 (*P*=0.17) (Figure 5).

## DISCUSSION

The findings of this study suggest good agreement for HEART scores between attending and resident physicians. Furthermore, the attending or resident years of experience did not have a significant impact on discrepancies in HEART scores. Thus, this study suggests that residents may be able to provide reliable HEART scores for patients who present to the ED. These findings are consistent with previous studies. For example, a 2022 retrospective review by Pawlukiewicz et al found fair to substantial agreement between emergency medicine and internal medicine attending and resident physicians in their HEART score assessments, indicating that resident assessments were generally reliable.^13^ However, the attendings and residents in the Pawlukiewicz et al study calculated HEART scores based on a retrospective medical records review rather than calculating scores immediately after patient encounters.

Studies comparing HEART scores between attending and resident physicians have indicated that despite minor variations, overall agreement is substantial. A 2021 study by Soares et al demonstrated an interrater agreement of 78% between attendings and residents, with the most common discrepancies occurring in the history component of the HEART score.^14^ Similarly, in 2019, Gershon et al found 84.2% agreement between attending and resident physician scores.^15^ Both studies support the reliability of resident assessments, particularly when standardized scoring forms are used. Another study found that the HEART score had a high predictive value for major adverse cardiac events across various levels of physician experience, underscoring its utility as a standardized tool in diverse clinical settings.^16^

Moreover, the implementation of standardized scoring forms has been shown to improve the consistency of HEART score assessments. For instance, Mahler et al found that using a standardized HEART Pathway not only improved agreement between physicians but also enhanced patient outcomes by reducing unnecessary testing, likely leading to substantial cost savings.^4^ The findings also imply a reduction in admissions and early discharges for low-risk patients. Another study indicated that structured protocols incorporating the HEART score could streamline decision-making processes and improve resource utilization in the ED.^3^

The improvement in scoring discrepancies for HEART scores ≤3 or >3 is critical for clinical decision-making. Standardized scoring forms help minimize subjective interpretations and ensure that all components of the HEART score are evaluated consistently. In 2021, Sevransky et al highlighted the importance of standardized assessment tools in reducing variability in clinical practice and improving patient outcomes.^17^ A standardized assessment tool is particularly important for patients with HEART scores around the threshold of 3, as the score determines whether a patient is discharged or requires further observation and workup.

### Limitations

Given the nature of this pilot study and limited sample size, larger studies carried out across multiple sites examining the reliability between HEART scores of attending and resident physicians are needed to determine if these results can be extrapolated to other physician populations.

## CONCLUSION

The agreement between attending and resident emergency medicine physicians, which improved following the use of a standardized scoring form, suggests resident physicians may be able to provide valid and reliable HEART scores. Future research should focus on multicenter trials to validate these findings across different hospital settings and explore the potential benefits of integrating HEART scores with other diagnostic tools to further refine risk stratification processes.

## Notes

*This article meets the Accreditation Council for Graduate Medical Education and the American Board of Medical Specialties Maintenance of Certification competencies for Patient Care, Systems-Based Practice, and Practice-Based Learning and Improvement.*
